# Childhood and Adolescent Cancer Care at a Tertiary Hospital in Northern Tanzania: A Retrospective Study

**DOI:** 10.1200/GO.22.00263

**Published:** 2023-06-29

**Authors:** Esther Majaliwa, Emily R. Smith, Cesia Cotache-Condor, Hannah Rice, Yotham Gwanika, Julia Canick, Nelson Chao, Kristin Schroeder, Henry E. Rice, Catherine Staton, Blandina T. Mmbaga

**Affiliations:** ^1^ Pediatric Hematology and Oncology Services, Kilimanjaro Christian Medical Centre, Moshi, Tanzania; ^2^ Kilimanjaro Christian Medical University College, Moshi, Tanzania; ^3^ Department of Emergency Medicine, Duke University Medical Center, Durham, NC; ^4^ Duke Global Health Institute, Duke University, Durham, NC; ^5^ Department of Surgery, Duke University Medical Center, Durham, NC; ^6^ University of North Carolina at Chapel Hill, Chapel Hill, NC; ^7^ Kilimanjaro Cancer Registry, Kilimanjaro Christian Medical Centre, Moshi, Tanzania; ^8^ Department of Medicine, Duke University Medical Center, Durham, NC; ^9^ Department of Oncology, Bugando Medical Centre, Mwanza, Tanzania; ^10^ Department of Pediatrics, Duke University Medical Center, Durham, NC; ^11^ Kilimanjaro Clinical Research Institute, Kilimanjaro Christian Medical Centre, Moshi, Tanzania

## Abstract

**PURPOSE:**

Over 400,000 children are diagnosed with cancer around the world each year, with over 80% of these children residing in low- and middle-income countries. This study aims to summarize the epidemiology and care patterns of newly diagnosed childhood cancer patients in Northern Tanzania.

**METHODS:**

Data from all children and adolescents (age 0-19 years) with newly diagnosed cancers were collected from the Kilimanjaro Cancer Registry located at the Kilimanjaro Christian Medical Centre. Descriptive and inferential analyses were used to compare the demographic and clinical characteristics of the participants over time, stage, and status at last contact. Statistical significance was set at *P* < .05. Secondary descriptive analysis was conducted on a subset sample with available staging data.

**RESULTS:**

A total of 417 patients were diagnosed with cancer between 2016 and 2021. There was an increase in the rate of patients with newly diagnosed pediatric cancer each year, particularly among children under age 5 years and 10 years. Leukemias and lymphomas were the leading diagnoses and accounted for 183 (43.8%) of all patients. Over 75% of patients were diagnosed at stage III or above. From a subset analysis of patients with available staging data (n = 101), chemotherapy was the most common treatment (87.1%), compared with radiotherapy and surgery.

**CONCLUSION:**

There is a significant burden of children with cancer in Tanzania. Our study fills crucial gaps in the literature related to the large burden of disease and survival for children with cancer in the Kilimanjaro region. Furthermore, our results can be used to understand the regional needs and guide research and strategic interventions to improve childhood cancer survival in Northern Tanzania.

## Teaser

There is a high burden of leukemias and lymphomas in Kilimanjaro, with over 75% of cases diagnosed at stages III-IV.

## INTRODUCTION

Each year, there are over 400,000 newly diagnosed children with cancer around the world, with over 80% of these children residing in low- and middle-income countries (LMICs).^1^ Treatment of pediatric cancer in LMICs is increasingly recognized as a growing global health burden although the focus on children's cancer care has only recently been prioritized in the global health agenda.^2,3^ In the past few years, several notable developments have highlighted the need to improve cancer care for children in LMICs. In 2017, the World Health Assembly Cancer Resolution called for resource-stratified guidance for cancer programs in LMICs, including interventions specifically for children.^4^ In 2018, the WHO Global Initiative for Childhood Cancer was announced at the United General Assembly, with a focus on increasing the survival rates of childhood cancers to 60% by 2030.^5^

**CONTEXT**

**Key Objective**
What are the epidemiologic patterns of childhood and adolescent cancer in the Kilimanjaro region?
**Knowledge Generated**
Leukemias and lymphomas were the leading diagnoses and accounted for 183 (43.8%) of all patients in the Kilimanjaro region. Over 75% of patients were diagnosed at stage III or above.
**Relevance**
Understanding the childhood and adolescent cancer burden and care patterns is an essential step to identify key elements for improvement across the entire continuum of care.

Many children with cancer in LMICs face barriers across the entire continuum of care, particularly for delays in diagnosis and initiation of treatment.^6^ Children often do not have access to high-quality care and have high rates of abandonment of treatment.^7^ These barriers lead to increased mortality risks, financial costs, and household impoverishment.^8-11^ Although recent progress has been made in cancer care in LMICs, survival rates in many regions remain far lower compared with those in high-income countries.^12^ We recently have shown that sub-Saharan African countries have the highest vulnerability of childhood cancer mortality associated with delays in care.^11^

Tanzania, a lower-middle–income country in sub-Saharan Africa, experiences many limitations in the access and resources to care for childhood cancers.^13^ There are only six local pediatric oncologists in the country, three of them are located in the urban capital city of Dar es Salaam, and one is located in the Northern region at the Kilimanjaro Christian Medical Centre (KCMC). Furthermore, there is only one pathologist per 1.8 million people.^14^ By contrast, the United States has one pathologist for every 20,638 habitants and approximately 2,000 pediatric oncologists.^14^

Studies on pediatric cancer care in Tanzania are scarce, with limited reports of the epidemiology and treatment capacity.^15-19^ The Kilimanjaro Cancer Registry (KCR) was recently extended to include children with cancer, and this resource can help assess the spectrum of cancer types, treatments, and burden in the region. The purpose of this study was to provide a summary of newly diagnosed child and adolescent patients with cancer between 2016 and 2021 through the KCR. In particular, we assessed cancer types, treatment use patterns, demographics, and clinical characteristics of children and adolescents with newly diagnosed cancers over this time period.

## METHODS

### Study Site

KCMC is a Zonal Referral Consultant Hospital for the Northern regions of Tanzania (Arusha, Dodoma, Kilimanjaro, Singida, and Tanga). KCMC houses one of the two cancer centers located in northern Tanzania, providing treatment for both pediatric and adult oncology patients. KCMC hosts the only pediatric oncologist in the region, as well as one pediatric surgeon and ophthalmology, orthopedics, and urology departments that care for children with cancers. Services provided include diagnosis, treatment, research, and outreach programs, with an onsite laboratory, 12 chemotherapy bays, 47 inpatient beds, and 4 consultation rooms. All conventional chemotherapy services are offered at KCMC and few targeted therapies through research grants. Radiation services are not currently offered, with patients being referred to other cities such as Dar es Salaam. Financial coverage for direct medical expenses and indirect expenses such as food, transportation, and education is supported by a range of resources at KCMC, resulting in no costs to pediatric oncology patients and their families. In addition to inpatient and outpatient cancer treatment services, KCMC offers home visits, palliative care, cancer screenings, and prevention and awareness programs within the greater Kilimanjaro region.

The KCR, created in 1998, is the oldest population-based cancer registry in Tanzania and a member of the African Cancer Registry Network (AFCRN).^20^ The KCR has included child and adolescent patients with cancer since 2016. Although children with cancer have been cared for at KCMC for many years, they were included within the KCR since 2016, when the specialized cancer care center for children was launched.

The KCR includes data from children across 14 hospitals among five districts in the Kilimanjaro region, including Moshi Urban, Moshi Rural, Rombo, Hai, and Siha. Continuous data collection by trained registry assistants located in each district of the catchment area provides key information to researchers, clinicians, and the Tanzanian Ministry of Health.

### Data Collection

Data from all children up to age 19 years with newly diagnosed cancer were collected from January 2016 through December 2021. We chose 2016 as a starting point because this is the year when KCMC started offering specialized oncology services for children. Demographic variables collected from the KCR included age at diagnosis (grouped into four categories, including 0-4, 5-9, 10-14, and 15-19 years), sex, and geographical region of referral. The geographical region was divided into the Kilimanjaro Region and Other Regions, and the latter included Manyara, Tabora, Mwanza, Dodoma, Simiyu, Mara, Geita, Tanga, Mbeya, Rukwa, Morogoro, Ruvuma, Singida, Kagera, Kigoma, Dar es Salaam, Shinyanga, Arusha, and neighbor country Kenya. Clinical characteristics included cancer diagnosis by group, treatment (surgery, chemotherapy, and/or radiotherapy), stage of cancer at diagnosis, and status (alive or dead) at last contact.

Cancer diagnosis was divided into 12 types according to the International Classification of Childhood Cancer, third edition: group I, leukemias, myeloproliferative diseases, and myelodysplastic diseases; group II, lymphomas and reticuloendothelial neoplasms; group III, CNS and miscellaneous intracranial and intraspinal neoplasms; group IV, neuroblastoma and other peripheral nervous cell tumors; group V, retinoblastoma; group VI, renal tumors; group VII, hepatic tumors; group VIII, malignant bone tumors; group IX, soft tissue and other extraosseous sarcomas; group X, germ cell tumors, trophoblastic tumors, and neoplasms of gonads; group XI, other malignant epithelial neoplasms and malignant melanomas; and group XII, other and unspecified malignant neoplasms.^21^

Stage of cancer at diagnosis was available for 101 of the 417 study participants. Although comparison of staging across childhood cancer types is complicated by the large number of staging systems used for individual cancer types in our data set (ie, TNM system for solid tumors, International Society of Pediatric Oncology system for Wilms Tumor, clinician judgment for hematologic malignancies, etc), we categorized all cancer types as stage I-IV by following the staging conversion methods available in the AFCRN *Childhood Cancer Staging Rules for Population Based Registries* manual.^22^ Given the recent inclusion of children in the registry, we were not able to collect specific information on the cause of death or long-term follow-up, such as 5-year survival rates.

### Data Analysis

Descriptive and inferential statistical analyses were performed to determine the distribution of diagnosis, treatment, status at last contact, and demographic information (full sample of 417 participants) and staging (subset sample of 101 participants for whom staging data were available). Chi-square statistics were calculated for categorical variables to assess for significance at *P* < .05. Statistical analyses were performed using STATA v15.1 (StataCorp, College Station, TX).

### Ethical Considerations

The study was approved by the KCMC Institutional Review Board (IRB) and the National Institute for Medical Research—Lake Zone Medical Research Coordinating Committee (Mwanza, Tanzania) and Duke University of Medicine IRB (Durham, NC; 45CFR46.101(b)).

### Patient Consent Statement

Consent was not obtained from each participant since it was not applicable. This study used data already available from a cancer registry, and no data were collected directly from participants through surveys or interviews. We confirm that the IRB waived the informed consent for this part of the data collection.

## RESULTS

We collected data on 417 pediatric and adolescent patients with cancer between 2016 and 2021 (Table 1). The leading age category in this cohort was under 5 years, making up to 36.2% (n = 151) of all patients. Across all years, the majority of patients were younger than 10 years (59.2%, n = 247), and the proportion of this age category increased from 50% in 2016 to 71% in 2021. Among all participants, 40% (n = 167) were located in the Kilimanjaro region and 60% (n = 250) traveled from other regions of Tanzania or neighboring countries. We found that 57.8% of the study sample were male and 42.2% were female. Of the three types of treatment reported, 77.2% (n = 322) of patients received chemotherapy, 17.4% (n = 70) of patients received surgery, and 7.9% (n = 28) of patients received radiotherapy. Some patients received a combination of treatments. The status at last contact reported that most patients in this cohort were alive (83.8%, n = 342). When stratifying by type of cancer, we found that group I (leukemias, myeloproliferative diseases, and myelodysplastic diseases) and group II (lymphomas and reticuloendothelial neoplasms) were the leading diagnoses and accounted for 43.8% (n = 183) of all patients.

**TABLE 1:**
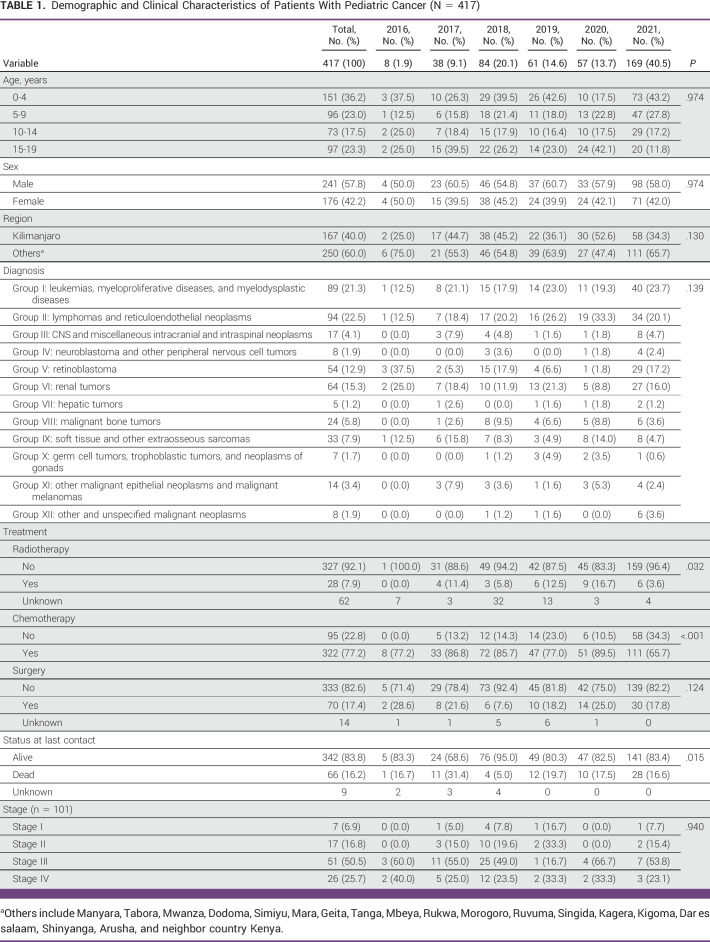
Demographic and Clinical Characteristics of Patients With Pediatric Cancer (N = 417)

**FIG 1:**
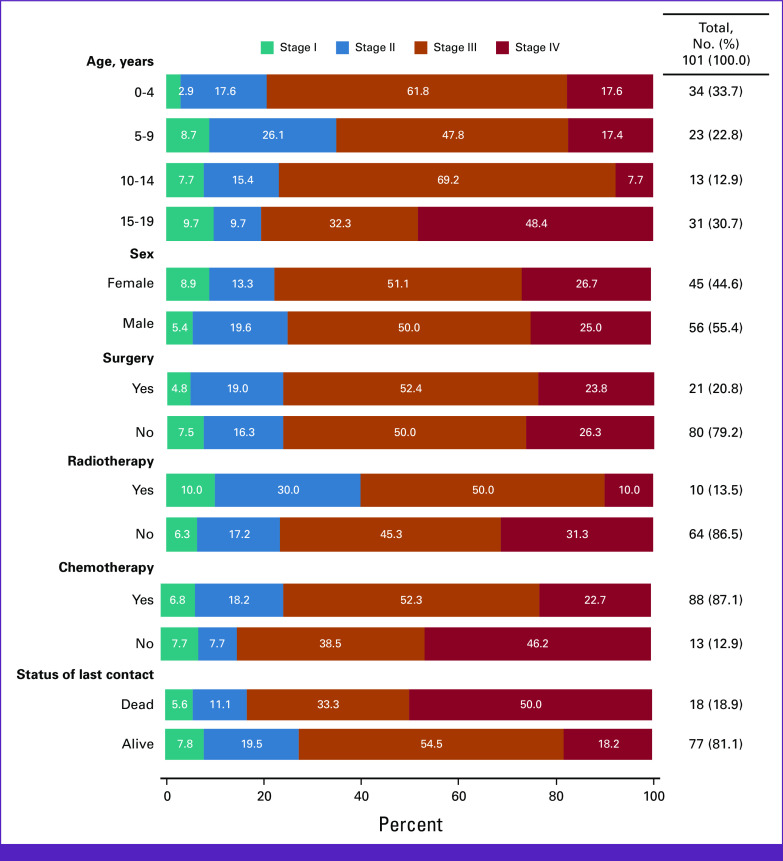
Distribution of demographic and clinical characteristics across stage at diagnosis (n = 101).

Stage of cancer at the time of diagnosis was available for 24.2% of the patients (n = 101; Table 2). From this subset, 7 (6.9%) patients were diagnosed at stage I, 17 (16.8%) at stage II, 51 (50.5%) at stage III, and 26 (25.7%) at stage IV. Among those patients diagnosed with stage III, 32 (62.8%) patients were younger than 10 years, and most of the patients diagnosed with stage IV (57.7%, n = 15) were between the age 15 and 19 years. Over 75% of the patients presented with higher stages of disease (stages III and IV). Patients age under 5 years and age between 10 and 14 years reported biggest percentages of stage III diagnoses ranging from 61.8% to 69.2%, respectively (Fig 1), whereas almost half of the patients age between 15 and 19 years presented with stage IV. However, patients under age 5 years included the highest proportion of cases diagnosed with stage III and above (79.4%). Around 83.3% of patients who died were diagnosed with stage III or above, and 72.7% of patients who lived were diagnosed with stage III or above. However, a half of patients (50%) who died were diagnosed with a stage IV and 54.5% of patients who lived were diagnosed with stage III.

**TABLE 2:**
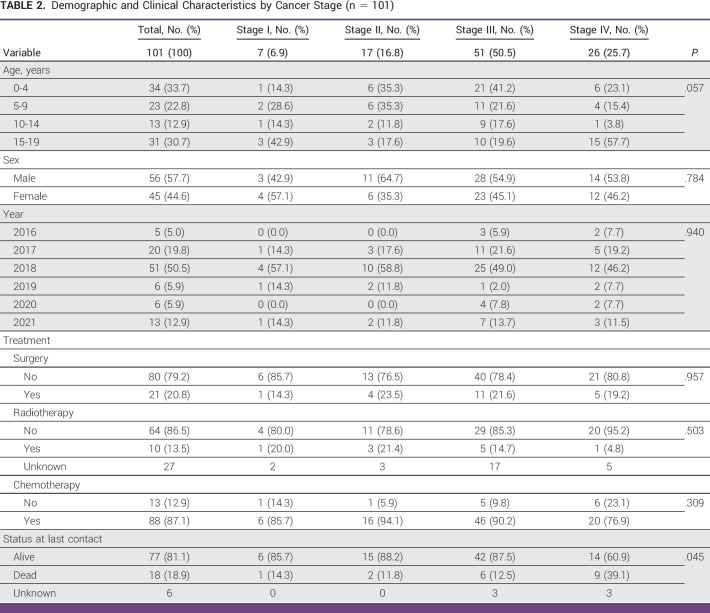
Demographic and Clinical Characteristics by Cancer Stage (n = 101)

**FIG 2:**
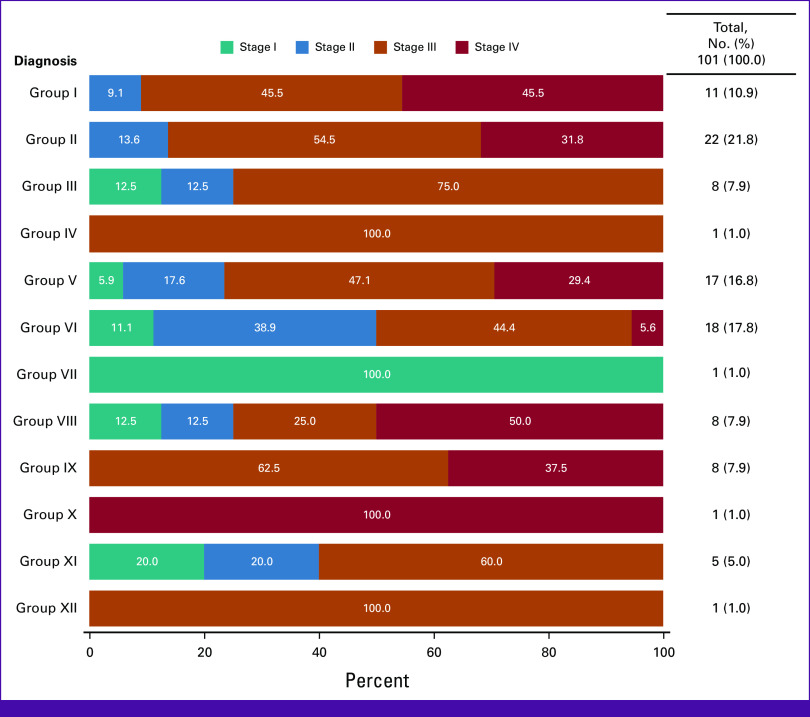
Distribution of type of cancer across stage at diagnosis (n = 101). Group I: leukemias and myeloproliferative diseases, myelodysplastic diseases; group II: lymphomas and reticuloendothelial neoplasms; group III: CNS and miscellaneous intracranial and intraspinal neoplasms; group IV: neuroblastoma and other peripheral nervous cell tumors; group V: retinoblastoma; group VI: renal tumors; group VII: hepatic tumors; group VIII: malignant bone tumors; group IX: soft tissue and other extraosseous sarcomas; group X: germ cell tumors, trophoblastic tumors, and neoplasms of gonads; group XI: other malignant epithelial neoplasms and malignant melanomas; group XII: other and unspecified malignant neoplasms.

**FIG 3:**
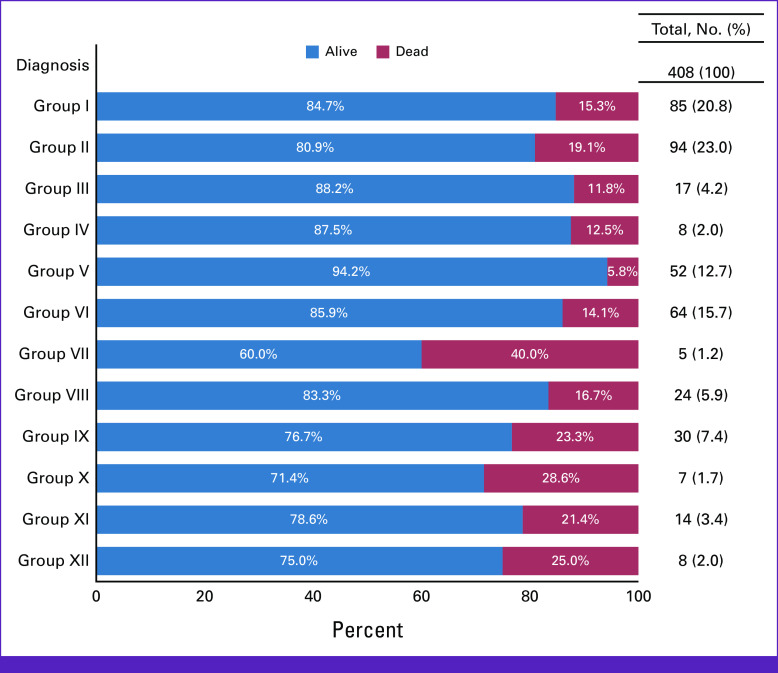
Distribution of type of cancer across patient's status at last contact (n = 408). Group I: leukemias, myeloproliferative diseases, and myelodysplastic diseases; group II: lymphomas and reticuloendothelial neoplasms; group III: CNS and miscellaneous intracranial and intraspinal neoplasms; group IV: neuroblastoma and other peripheral nervous cell tumors; group V: retinoblastoma; group VI: renal tumors; group VII: hepatic tumors; group VIII: malignant bone tumors; group IX: soft tissue and other extraosseous sarcomas; group X: germ cell tumors, trophoblastic tumors, and neoplasms of gonads; group XI: other malignant epithelial neoplasms and malignant melanomas; group XII: other and unspecified malignant neoplasms.

When stratifying by type of cancer and staging, 100% (n = 1) of the patients in group IV and group XII were diagnosed at stage III and 75% (n = 6) of patients in group III (CNS and miscellaneous intracranial and intraspinal neoplasms) were diagnosed at stage III (Fig 2). Also, 100% (n = 1) of patients from group X (germ cell tumors, trophoblastic tumors, and neoplasms of gonads) were diagnosed with stage IV, whereas 100% (n = 1) of patients from group VII (hepatic tumors) were diagnosed with stage I. Among patients from group I and group II (leukemia and lymphoma), 45.5%-54.5% of patients were diagnosed with stage III, respectively, and 31.8%-45.5% of patients were diagnosed at stage IV, respectively. When stratifying by type of cancer and status at last contact, over 60% of patients from across all diagnoses were reported as alive (Fig 3). The highest percentage of deaths (40%) were reported among patients from group VII (hepatic tumors).

## DISCUSSION

This study offers one of the most comprehensive overviews of the epidemiology and treatment patterns of children in Northern Tanzania using a robust and standardized cancer registry. Our findings suggest an increase in the rate of patients with newly diagnosed pediatric cancer over recent years, particularly among children under age 5 years and 10 years. Leukemias and lymphomas were identified as the leading diagnoses, and over 75% of patients were diagnosed at stage III or above. Understanding the childhood cancer burden and care patterns is an essential step to identify key elements to improve the care of children with cancer across the entire continuum of care.

There are several potential reasons behind the recent increase in newly diagnosed patients with cancer. First, we believe that more families each year might have decided to bring their child to receive treatment at KCMC as the specialized children's cancer center has become well known in the region. Similarly, the addition of the only pediatric oncologist in the region might have led to increased rates both of referrals of children previously not cared for within cancer systems, and children who would not have been able to access diagnosis and care at all. However, given the lack of known cancer burden in the region, it is unclear if the actual patient rate changed over time or if this simply reflects increased recognition of the patient burden. Another factor that might have contributed to the increased patient number may be related to decreased financial strain on patients. Out-of-pocket expenses for health care is a known risk factor for impoverishment and a driver of cancer mortality, especially in LMICs.^11,23,24^ In 2021, KCMC eliminated out-of-pocket expenditures and instituted a full-coverage system for all pediatric patients with cancer.^25^ The new pediatric cancer facility provides free food, transportation, education for all children and a soon-to-open lodging for families who may experience financial barriers to accessing care. Reducing out-of-pocket health care expenses and indirect expenses might have led to an increase in a family's ability to seek care for their children, thereby improving access to diagnosis and reducing treatment abandonment.

The highest number of newly diagnosed cancers was found among patients younger than 5 years. Most of these children attend local pediatric clinic and vaccination appointments, which may favor timely diagnosis and referral.^26^ However, our study also found that children under age 5 years presented with higher stages of disease compared with other age groups. This finding is consistent with the recent Global Burden of Disease Childhood Cancer study, which found that the greatest burden of cancer measured in disability-adjusted life years (DALYs) was among young children age 0 to 4 years.^27^ In addition, this high DALY burden was driven by years of life lost (ie, mortality) rather than years of life lived with disability, suggesting that the risk of mortality is substantial for the youngest children. These findings suggest that timely referral, diagnosis, and treatment should be part of the country's health priority package for young children.

We found a high burden of leukemias and lymphomas across all age groups and all years in our study. Other cancer centers in Tanzania and other African countries such as Sudan, Kenya, and Rwanda also reported a high burden of hematologic malignancies.^28^ Globally, leukemias constitute the highest childhood cancer burden, with 34% of all childhood cancer DALYs attributable to leukemias solely.^27^ Non-Hodgkin lymphomas, including Burkitt's lymphomas, has the highest burden in sub-Saharan Africa than anywhere else in the world, responsible for up to 16.5% of all childhood cancers and up to half of all childhood cancers in tropical African countries.^27,29^ Burkitt's lymphomas are relatively easy to diagnose as they present as aggressive and fast-growing tumors. However, the aggressive growth of hematologic cancers in children also highlights the need for timely treatment and systems to minimize abandonment of care. In poor families, particularly in rural areas, lengthy treatment regimens are a major financial and geographic obstacle to seeking and maintaining patients with cancer. Thus, packages of childhood cancer care in LMICs should take into account these barriers to ensure swift diagnosis and treatment for fast-growing cancers such as Burkitt's lymphomas.

We found a relatively high rate of children presenting with advanced-stage disease, with over 75% of patients with cancer diagnosed at stages III and IV. Similar trends have been reported in other studies from Africa.^30,31^ These patterns in LMICs have been explained by complex referral systems, in which children with cancers are first seen at local health clinics and cannot be evaluated at district or national centers without a clinician referral. By the time these patients are referred to a higher-level hospital, they are often already in an advanced stages of disease. Some cancer symptoms can be confused with other common pediatric illnesses like dengue, typhoid, flu, chickenpox, pneumonia, and diarrhea.^32^ This often leads to misdiagnosis and a wrong path of treatment. Consequently, these patients have usually been seen at district and regional hospitals by the time they are referred to the specialized oncology centers. However, there is also the possibility that patients present late because of barriers in seeking timely care. For instance, community stigma, cultural beliefs, and traditional medicine are common determinants of delayed care in other low-resource settings.^33-36^ Further data collection is needed to determine the reasons why children with cancer are diagnosed at late stages in the Kilimanjaro region.

The patients at KCMC were more likely to receive chemotherapy, compared with radiation therapy or surgery. This pattern aligns with the high proportion of hematologic malignancies reported in our cohort and is similar to treatment patterns seen in many LMICs.^37^ However, many children's malignancies such as neuroblastomas, osteosarcomas, and Wilms tumor require multimodality care, and lack of radiation therapy and surgery constitutes a major challenge to comprehensive and high-quality cancer care. There is a shortage of access to radiation therapy in many LMICs, which might contribute to the observed pattern of care in Tanzania.^38^ The shortage of pediatric surgical workforce in many African countries may play a similar role in the care pattern at KCMC.^39^ Several other factors might contribute to these care patterns, including a potential cultural fear of surgery and a preference for traditional medicine over allopathic medicine that especially disfavors radiotherapy.^40,41^ In addition, medicines are often used for several other conditions widely prevalent in Africa, such as malaria and tuberculosis.^42,43^ This widespread use could have normalized the perception of medical treatments and help parents become more comfortable with the notion of their children undergoing chemotherapy.

Currently, long-term follow-up of children in the KCMC cancer registry remains lacking. As Piñeros et al suggested, although overall and cancer-related mortality is an essential metric of cancer care, it can be difficult to be tracked in many LMICs because of a multitude of data collection barriers.^44^ Examples of barriers are the absence of vital statistics and poor data quality.^45,46^ Children with cancer must overcome multiple barriers to reach the health system and be included in a registry.^37^ Breaks in this chain may occur at any step, generating significant gaps in data.

To our knowledge, our study is the most comprehensive overview of the epidemiology and care of childhood and adolescent cancer care in the Kilimanjaro region, using a hospital-based cancer registry. However, some limitations warrant discussion. First, child and adolescent data were only available from 2016 despite the fact that KCR is the oldest population-based registry in Tanzania. This limited our capacity to calculate survival rates and to perform survival analyses over long treatment periods. Second, data availability and staging remain a challenge, especially in low resources. Staging data were available for <25% of our cohort and were recorded using several systems according to the type of diagnosis. In addition, we used a stage I to stage IV system in line with the conversions suggested by the AFCRN *Childhood Cancer Staging Rules for Population-Based Registries* manual.^22^ This guideline recommends the use of a two-tiered approach for staging definitions to offer an alternative option in a context of limited data availability by providing with the lower tier on the basis of a less detailed criteria. The main purpose of this simplified staging system, particularly for the complex and varied staging systems used for childhood cancers, is to serve as a practical tool to assess epidemiologic analysis of trends of presentation of patients with childhood cancer at the population level. This staging system for registry purposes is not intended to replace clinical staging systems or to determine the treatment and/or prognosis of individual patients. Finally, despite the limitations that a cross-sectional model can offer in terms of causal inference and data availability; we have enhanced this study by using data from a validated population-based registry.

In conclusion, our study supports the need to enhance public health efforts on childhood and adolescent cancer care in Tanzania for children with cancer. The KCMC registry provides vital insights into current diagnostics, treatments, and outcome patterns for patients with pediatric cancer in the Kilimanjaro region. Earlier identification of childhood cancers may improve overall outcomes among this population. Given the increasing number of pediatric patients with cancer in Tanzania, understanding the current landscape of cancer needs and models of care is an essential step toward the scaling up of health systems and the improvement of survival rates in the Kilimanjaro region.
